# A Lightweight Strip Steel Surface Defect Detection Network Based on Improved YOLOv8

**DOI:** 10.3390/s24196495

**Published:** 2024-10-09

**Authors:** Yuqun Chu, Xiaoyan Yu, Xianwei Rong

**Affiliations:** 1 School of Computer Science and Information Engineering, Harbin Normal University, Harbin 150025, China; chuyuqun@stu.hrbnu.edu.cn; 2 School of Physics and Electronic Engineering, Harbin Normal University, Harbin 150025, China; yuxiaoyan@hrbnu.edu.cn

**Keywords:** defect detection, YOLOv8, lightweight, attention mechanism

## Abstract

Strip steel surface defect detection has become a crucial step in ensuring the quality of strip steel production. To address the issues of low detection accuracy and long detection times in strip steel surface defect detection algorithms caused by varying defect sizes and blurred images during acquisition, this paper proposes a lightweight strip steel surface defect detection network, YOLO-SDS, based on an improved YOLOv8. Firstly, StarNet is utilized to replace the backbone network of YOLOv8, achieving lightweight optimization while maintaining accuracy. Secondly, a lightweight module DWR is introduced into the neck and combined with the C2f feature extraction module to enhance the model’s multi-scale feature extraction capability. Finally, an occlusion-aware attention mechanism SEAM is incorporated into the detection head, enabling the model to better capture and process features of occluded objects, thus improving performance in complex scenarios. Experimental results on the open-source NEU-DET dataset show that the improved model reduces parameters by 34.4% compared with the original YOLOv8 algorithm while increasing average detection accuracy by 1.5%. And it shows good generalization performance on the deepPCB dataset. Compared with other defect detection models, YOLO-SDS offers significant advantages in terms of parameter count and detection speed. Additionally, ablation experiments validate the effectiveness of each module.

## 1. Introduction

As one of the important industrial materials, strip steel inevitably produces defects such as cracks, spots, and scratches during processing. These defects affect the appearance and quality of the products, thereby reducing corporate profits [1]. Therefore, achieving a lightweight and high-precision strip steel surface defect detection algorithm is of great significance for improving the surface quality of strip steel products.

Surface defect detection has traditionally depended on manual visual inspection, a method that is both labor-intensive and time-consuming. This approach is inherently flawed because of the variability in individual skills and experience, which can lead to inconsistent results, including both misdetections and missed detections. However, the advent of machine vision technology has introduced more efficient and reliable alternatives. A major milestone in this transition was reached in 1983 when Honeywell in the United States developed a surface defect detection device utilizing charge-coupled device (CCD) technology [2]. Subsequently, various defect detection algorithms have been developed. For instance, Guo et al. [3] introduced an edge detection algorithm that combines Kirsch and Canny operators, which has proven effective in detecting defects such as bubbles and pits on ceramic surfaces. Similarly, Nieniewski et al. [4] developed a system based on morphological operations to extract features associated with orbital defects. However, traditional machine vision algorithms lack consistent robustness and generalization capabilities across diverse defect detection scenarios, making it difficult to meet the requirements of defect detection. With the development of deep learning, defect detection models based on deep learning have become a current research hotspot. Deep learning-based object detection algorithms are generally classified into two-stage and single-stage detection approaches. Two-stage algorithms include Faster R-CNN [5] and Mask R-CNN [6], while single-stage algorithms include YOLO [7,8,9,10,11,12], SSD [13], and EfficientDet [14]. Among them, the application of YOLO in defect detection has been steadily growing because of its outstanding performance. While YOLO offers a quicker detection speed compared with two-stage algorithms, its detection accuracy still needs improvement when dealing with complex and diverse steel defects.

To improve defect detection accuracy, this paper proposes a lightweight strip steel surface defect detection network based on an improved YOLOv8. Our proposed method significantly reduces the parameter count while enhancing accuracy, effectively improving the efficiency of strip steel surface defect detection. The contributions of this study are as follows:

1. We utilized the lightweight network module StarNet, which significantly reduces the parameter quantity, enhances the multi-scale feature extraction capability, and improves detection accuracy.
2. We combined the DWR module with the C2f module, enabling the model to capture features across various scales more effectively while maintaining high computational efficiency and network depth.
3. We introduced the occlusion-aware attention mechanism SEAM into the detection head, improving the model’s capability to capture and process features of occluded objects.

The structure of this paper is as follows: Section 2 provides an overview of the existing YOLO algorithms and related work. Section 3 introduces the improved strip steel surface defect detection network. Section 4 presents the dataset, experimental process, and results. Finally, Section 5 summarizes the main contributions of this study.

## 2. Related Work

Flaw detection approaches are primarily categorized into traditional machine learning techniques and deep learning methods.

### 2.1. Traditional Machine Learning Methods

Early methods for metal defect detection using computer vision largely relied on hand-crafted features. Image preprocessing was used to enhance image quality and reduce noise, while feature extraction captured critical information from the images using manually designed features. Specific classification algorithms were then employed to detect and identify defect areas in the images. Machine learning techniques played a crucial role in this process by automatically extracting image features and classifying them into different categories. For instance, Local Binary Pattern (LBP) [15,16] and Histogram of Oriented Gradients (HOG) [17] were widely applied to extract texture features effectively, demonstrating strong robustness in traditional computer vision tasks. At the same time, classifiers such as Support Vector Machines (SVMs) and Decision Trees [18] were commonly used to classify defects based on the extracted features. Zhang et al. [19] introduced a technique that combines Gaussian functions fitted to histograms with a membership matrix of test images to identify and locate defects. By utilizing machine learning techniques, traditional manual detection methods were replaced, significantly improving the efficiency of defect detection. Machine learning algorithms can automatically analyze and process large amounts of data, not only enhancing detection accuracy but also greatly speeding up the detection process. This reduces human error and makes defect detection more intelligent and efficient.

### 2.2. Deep Learning Methods

As deep learning has advanced rapidly, Convolutional Neural Networks (CNNs) have been increasingly utilized in object detection tasks.

Two-stage methods are known for their high precision in detecting surface defects. For instance, Jiao et al. [20] created a complete detection system by integrating anchor-free technology with Fast R-CNN, designed to detect defects on crop surfaces. Similarly, Cha et al. [21] enhanced Faster R-CNN by altering the Region Proposal Network (RPN) within ZFNet. Hou et al. [22] introduced a Cascade Mask R-CNN with a transfer learning approach for detecting cable defects. While these methods excel in accurately identifying various defects, their low detection speed remains a significant limitation for industrial applications.

Compared with two-stage detectors, single-stage detectors enhance the speed of detection by treating object detection as a regression problem, directly estimating anchor points on the feature map where objects might be located [23]. Xing et al. [24] improved YOLOv3 by integrating additional prediction layers to identify railway surface imperfections. Li et al. [25] developed an enhanced YOLOv4 algorithm that integrates CBAM and RFB for detecting strip steel surface defects. Ying et al. [26] created a YOLOv5s-based model to detect defects in steel wire braided hoses, particularly targeting ultra-small defects by adding a larger-scale prediction layer. Li et al. [27] developed a detection mechanism grounded in YOLOv5, employing optical correction and patching techniques for insulator defect identification. Gao et al. [28] developed a new window-shifting strategy for the Swin Transformer to enhance feature extraction capabilities. This strategy improves the model’s capability to capture spatial relationships while boosting both accuracy and efficiency, making it ideal for applications such as object detection and image segmentation. Guo et al. [29] combined YOLOv5 with Transformer modules to expand the receptive field for precise surface defect prediction. Generally, single-stage methods can incorporate additional modules to achieve rapid detection speeds, but they often face challenges with minor and blurry defects. While one-stage methods boost detection speed, their accuracy needs enhancement. In July 2022, Alexey Bochkovskiy et al. introduced YOLOv7. YOLOv7 uses an Efficient Layer Aggregation Network (ELAN), which increases detection accuracy through the extensive stacking of computational blocks while maintaining fast inference speed. In 2023, YOLOv8 was introduced, which retains the YOLOv5 backbone structure but replaces the C3 module with the C2f module, providing a richer gradient flow. The C2f module is lighter, has stronger feature fusion capabilities, and accelerates inference speed.

Although single-stage detectors offer faster detection speed compared with two-stage detectors, the parameter count and computational complexity of standard detection models are still too high to meet real-time requirements in resource-constrained environments such as mobile devices and embedded systems. Therefore, it is necessary to design lightweight networks to balance computational efficiency and detection accuracy. Existing lightweight networks include the YOLO series, MobileNet series, EfficientDet series, among others. The lightweight versions of the YOLO series include YOLOv3-tiny, YOLOv4-tiny, YOLOv5s, YOLOv7-tiny, and YOLOv8n. These versions minimize the model’s computational cost and parameter count through optimizations in network structure, loss functions, data augmentation strategies, and feature fusion modules. The MobileNet series (V1, V2, V3) [30,31,32], proposed by Google, are lightweight convolutional neural networks that significantly reduce the computational and parameter overhead using techniques like depthwise separable convolutions, inverted residuals, and automated search optimization, while progressively improving model representation and accuracy across different versions. The EfficientDet series (D0–D3), introduced by Google in 2020, are efficient object detection models based on the EfficientNet backbone, achieving a good balance between detection accuracy and efficiency through the compound scaling strategy and BiFPN (Bidirectional Feature Pyramid Network) design. EfficientDet-D0 is the most lightweight version in the series, suitable for scenarios with extremely limited resources, with a low parameter count and relatively high detection accuracy. EfficientDet-D1 to D3 strike a good balance between model complexity and accuracy, making them suitable for real-time applications with high accuracy requirements, such as drone object detection and video surveillance. Although the existing lightweight networks have achieved a balance between accuracy and efficiency, they still have limitations in applicable scenarios and struggle with detecting small objects accurately. Therefore, this paper proposes a new lightweight object detection network. Drawing inspiration from YOLOv8n, we introduce a model called YOLO-SDS, aimed at boosting both accuracy and detection speed. In comparison with the original YOLOv8n, YOLO-SDS enhances prediction precision, significantly decreases the parameter quantity, and can detect a diverse array of defect types.

## 3. Proposed Method

**Figure 1:**
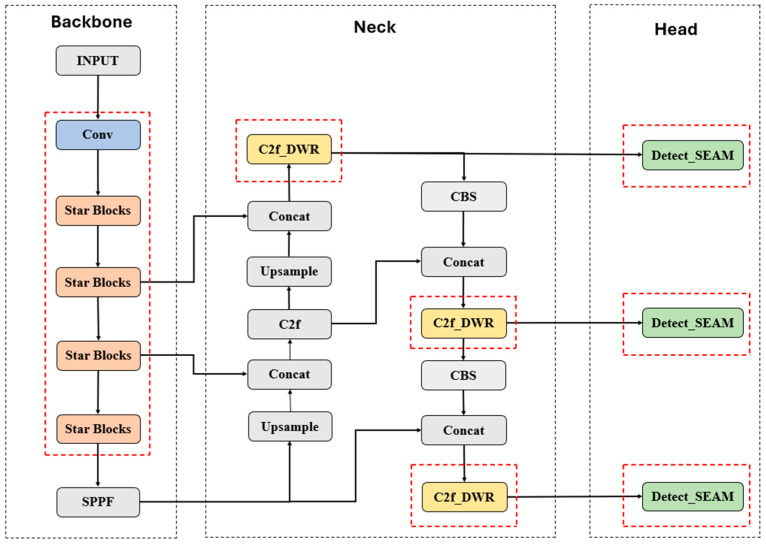
Architecture of the proposed YOLO-SDS.

To tackle the challenges of detecting strip steel surface defects, we propose a detection network specifically designed for this purpose, based on YOLOv8, named YOLO-SDS. The suggested network architecture is highlighted in Figure 1. The model first replaces the backbone part of the YOLOv8 network with StarNet [33]. StarNet has high-dimensional feature processing capabilities and can map inputs to a high-dimensional nonlinear feature space through Star Operations without increasing the network width. This approach is similar to kernel methods but avoids actually increasing network complexity, thus achieving efficient data processing. Secondly, we introduce the DWR [34] module combined with the C2f module of YOLOv8, expanding the receptive field without increasing computational complexity and maintaining the stability of network training. Finally, the traditional detection head (Detect) in YOLOv8 is replaced with Detect_SEAM [35], enhancing feature representation, improving model performance, and increasing the model’s generalization ability.

### 3.1. StarNet Module

Because of the varying sizes of surface defects on steel and the fact that some defects are extremely subtle, the captured images are often blurry. This causes the backbone of the YOLOv8 model to struggle to extract features effectively during detection, hindering the distinction of the edges, textures, and shapes of the defects and resulting in low detection efficiency. To address this issue, we introduced the StarNet module to replace the original backbone of YOLOv8. StarNet is a novel and efficient network architecture that primarily leverages the “Star Operation” to achieve effective feature mapping. The Star Operation refers to the element-wise multiplication of features from two different feature spaces. Its core working principle lies in mapping the input into a high-dimensional, nonlinear feature space through this element-wise multiplication, similar to the kernel trick in traditional machine learning. The advantage of the Star Operation is its ability to enhance the dimensionality of the feature space significantly without increasing the network width (number of channels). Specifically, in a single-layer network, the Star Operation is typically represented as follows:

(1) $${(W}_{1}^{T}X+B_{1})⊙{(W}_{2}^{T}+B_{2})$$

where $W_{1}^{T}$ and $W_{2}^{T}$ are two weight matrices, X represents the input features, and ⊙ denotes the element-wise multiplication operation.

**Figure 2:**
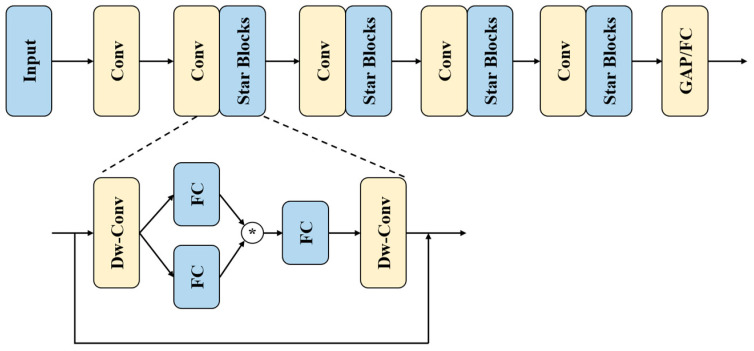
StarNet architecture overview.

StarNet is an efficient neural network built around the core concept of the Star Operation, characterized by its simplicity and powerful performance. StarNet employs a layered network architecture, typically divided into four stages, with the number of feature channels gradually increasing at each stage. Each stage consists of a Convolutional Layer (Conv Layer) and a Star Operation Module (Star Block). The Conv Layer is responsible for down-sampling the input features (reducing resolution) while increasing the number of channels. The Star Operation Module includes a Depthwise Convolution Layer (DW-Conv), Fully Connected Layer (FC), activation function (ReLU6), and the Star Operation. The DW-Conv layer is used for independent convolution operations on each channel, maintaining spatial resolution. The FC layer maps the input features to different subspaces. ReLU6 introduces non-linearity, enhancing the model’s representational capacity. The Star Operation, as the core operation, fuses features from two branches through element-wise multiplication. The StarNet framework is illustrated in Figure 2.

### 3.2. C2f-DWR Module

Although YOLOv8 shows outstanding results in detection speed and accuracy, it still has limitations when handling the contextual elements in the images of steel surface imperfections. Because of the large variations in defect sizes, with some being very subtle, the model needs to capture multi-scale contextual information effectively during detection. Additionally, YOLOv8’s original feature extraction module may struggle to extract detailed information fully when faced with complex textures and edges, which can negatively impact detection accuracy. Moreover, in deep networks, the vanishing gradient problem may make the model difficult to train effectively. Therefore, YOLOv8 requires improvements in multi-scale information extraction and training stability in deep networks. To address these issues, the DWR module was introduced into YOLOv8. The DWR module, with its unique method for acquiring multi-scale contextual information, can significantly enhance YOLOv8’s ability to extract features across different scales.

**Figure 3:**
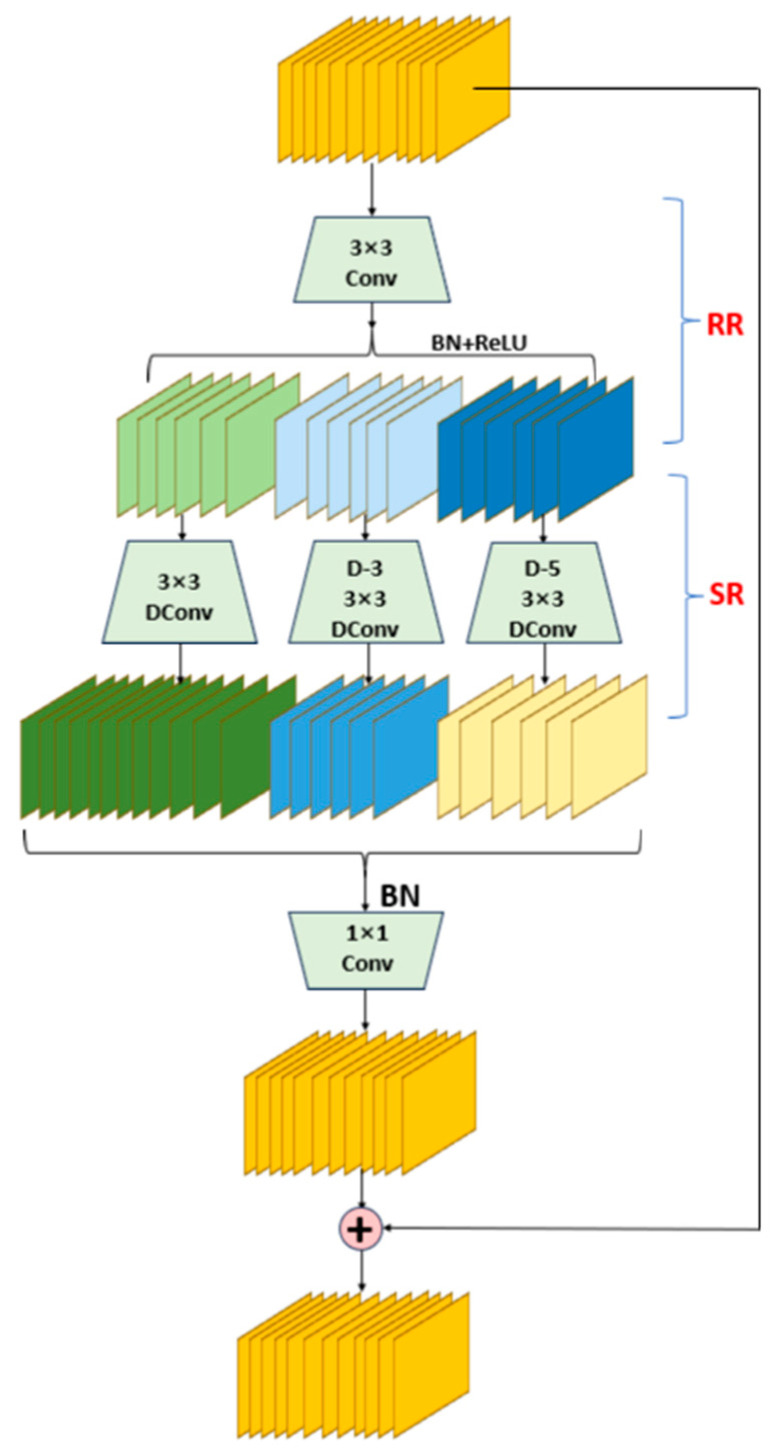
Diagram of the DWR module structure.

The working principle of the DWR module is divided into two stages. The first stage is Region Residualization. In this stage, the input feature maps are processed by a 3 × 3 convolution layer, a batch normalization (BN) layer, and a ReLU activation function to generate concise feature maps with regional forms. These feature maps are prepared for morphological filtering in the second stage. Unlike traditional multi-scale feature extraction methods, Region Residualization simplifies the expression of feature maps, making them more amenable to subsequent convolution operations. The second stage is Semantic Residualization. In this stage, deep convolutions with different dilation rates are applied to the concise feature maps for morphological filtering. Each convolution operation employs a specific receptive field size to process different groups of feature maps, ensuring the diversity and completeness of the feature representations. In this manner, the DWR module can efficiently extract multi-scale contextual information from the feature maps, avoiding the computational waste and learning difficulties caused by the redundant receptive fields in traditional methods. The structure of the DWR module is illustrated in Figure 3.

**Figure 4:**
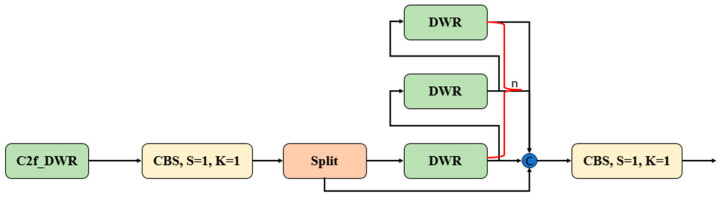
Diagram of the C2f_DWR structure.

In the original YOLOv8, the C2f section contains multiple Bottlenecks. The Bottleneck structure primarily focuses on extracting local features. Although it is computationally efficient, it has certain limitations in capturing multi-scale contextual information. In contrast, the DWR module, by introducing various dilated convolutions and integrating multi-scale features, can more comprehensively capture contextual information at different scales. Therefore, we replaced the original C2f module with the C2f_DWR module to enhance the model’s capability for multi-scale feature extraction. The C2f_DWR structure diagram is shown in Figure 4.

### 3.3. SEAM Module

The production environment of strip steel is complex, and occlusion has consistently been a challenge in steel defect detection. Finished strip steel is often partially obscured by other products, making it difficult for models to detect and identify defects fully. Traditional object detection methods struggle to capture the complete features of occluded objects in such scenarios, resulting in missed or false detections. Moreover, in complex environments, the mutual occlusion between background and foreground objects increases the risk of misjudgment by the model, affecting overall detection accuracy and robustness. Therefore, we introduced the Separated and Enhancement Attention Module (SEAM) module into the detection head of YOLOv8 to amplify the model’s skill in detecting targets in complex occlusion scenarios and improve its feature extraction of occluded objects.

**Figure 5:**
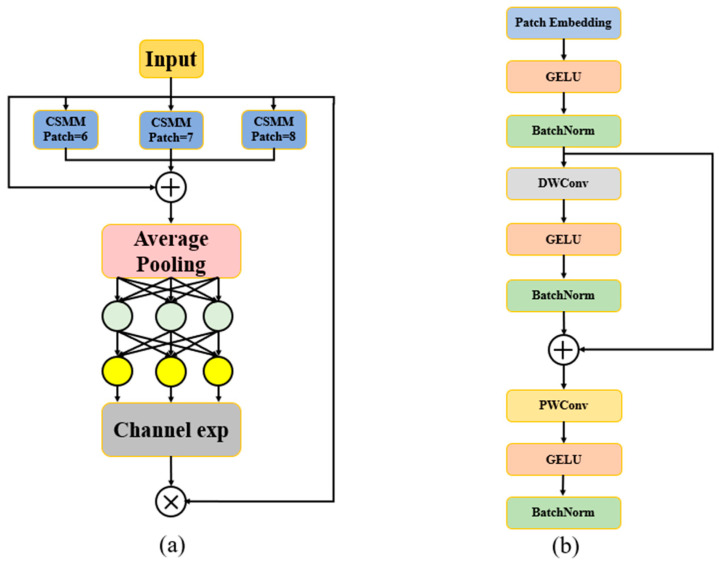
Illustration of SEAM. (**a**) The architecture of SEAM. (**b**) The structure of CSMM.

As shown in Figure 5, the SEAM module begins with the input layer, which receives feature inputs from the previous layer of the network. These features are then fed into the CSMM module for enhancement and processing. The CSMM module processes the feature map through a series of operations, including Patch Embedding, GELU activation function, Depthwise Convolution, and Pointwise Convolution, to enhance the feature representation capability of the image. Different CSMM modules extract features at various scales based on different patch sizes. This design allows the SEAM module to capture multi-scale features of the image effectively. The multi-scale features extracted from different CSMM modules are weighted and aggregated, enriching the feature representation of the image. The aggregated features are then subjected to average pooling to further reduce the feature dimensions. The pooled features are input into a two-layer fully connected network to learn the weight relationships between channels. Subsequently, channel expansion is performed to enable the model to better differentiate the importance of different channels and to further amplify the response to occluded areas. Finally, the result of channel expansion is used as attention weights and multiplied with the original feature map to obtain the final output feature map. This process enhances the focus on occluded regions and reduces the influence of background areas.

**Figure 6:**
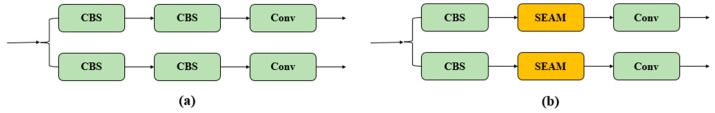
(**a**) Original YOLOv8 detection head; (**b**) Proposed detection head in this paper.

By integrating the SEAM module into the detection head of YOLOv8, the model’s ability to handle occluded objects is significantly improved, and the precision of feature extraction is optimized. Specifically, the occlusion-aware mechanism can actively identify areas in the image that are likely to be occluded, adjusting the network’s feature-capturing functionality process for these regions and allowing the network to better understand the details and characteristics of these complex areas. By enhancing the feature representation of these regions, the SEAM module effectively prevents information loss from occluded objects, ensuring that the model can more completely and accurately capture the entire target. The structure of the SEAM detection head is shown in Figure 6.

## 4. Experimental Results and Analysis

### 4.1. Dataset

We validated the effectiveness of YOLO-SDS using the NEU-DET dataset, which contains the following six types of steel surface defects: cracks (crs), inclusions (ins), patches (pas), pitted surfaces (pss), rolled-in scale (rs), and scratches (scs). Each defect type consists of 300 samples, with each image sized at 200 × 200 pixels. The NEU-DET dataset is allocated between training and testing sets with a 9:1 distribution, resulting in 1620 images for training and 180 images for testing.

### 4.2. Experimental Setup

We carried out our experiments on a machine using Ubuntu 22.04.3, with the experimental environment based on PyCharm 2024.2.1 software. Python 3.9.18 was the programming language utilized, and PyTorch 1.13.1 served as the deep learning framework. The CPU was a 13th Gen Intel (R) Core (TM) i5-13400F × 16, running at a frequency of 3.0 GHz, and the GPU was an NVIDIA GeForce RTX 4060 Ti with CUDA version 11.7. The hyperparameters used during training were as follows: The setup included a starting learning rate of 0.01, a weight decay factor of 0.0005, and a momentum value of 0.937. The size of the input images in all experiments was set to 640 × 640 pixels. During training, the epoch count was established at 200, with a batch size of 16, and the other parameters were kept at the default settings of the YOLOv8n model.

### 4.3. Performance Assessment

It is crucial to emphasize both accuracy and speed during the detection process. Therefore, we use precision, recall, F1 Sscore, mAP@0.5, Params, and FLOPs as the seven metrics to evaluate the model’s performance. Precision refers to the ratio of correctly identified samples to the overall sample count, serving as a measure of the model’s classification accuracy. The recall score reflects the percentage of real positive cases that are properly classified as positive, reflecting the model’s capability to detect all positive instances. The F1 score is a comprehensive performance metric, calculated from precision and recall, taking both into account. mAP is used to evaluate the accuracy of object detection algorithms across different categories. mAP@0.5 represents the mean average precision when the IoU threshold is set to 0.5. Params reflect the number of parameters that need to be learned in the model, usually related to the model’s complexity. The computational complexity of a model is evaluated using FLOPs (Floating Point Operations Per Second), which reflect the number of floating-point operations conducted in inference. Higher FLOPs values reflect greater computational demands, often correlating with improved performance, accuracy, and increased resource consumption. FPS (Frames Per Second) is an important metric that reflects the inference efficiency and real-time performance of a model. It indicates the number of image frames the model can process per second. These metrics comprehensively consider the model’s accuracy, efficiency, and computational complexity, providing a thorough quantitative evaluation of the improvements made to the model. The measurement equations are shown in Equations (2)–(5).

(2) $$Precision=\frac{TP}{TP+FP}$$

(3) $$Recall=\frac{TP}{TP+FN}$$

(4) $$AP=\int_{0}^{1}P(r)dr$$

(5) $$mAP=\frac{1}{\;k}\sum_{k}^{i}AP$$

where TP represents the count of defect samples accurately detected; FP is the number of detected non-defect samples; FN is the number of defect samples detected incorrectly; and P and R represent precision and recall.

### 4.4. Experiments and Results

**Figure 7:**
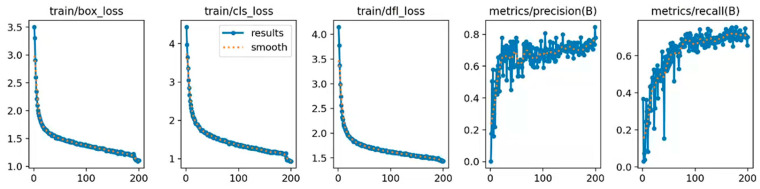
Loss curve of YOLO-SDS.

To assess the performance of the YOLO-SDS algorithm more effectively, we examined the outcomes of its training. During training, we set the number of epochs to 200. Figure 7 shows the loss curve of YOLO-SDS during training. As shown in Figure 7, the loss value consistently decreases and eventually stabilizes over 200 training epochs, positively impacting the model’s prediction accuracy.

**Figure 8:**
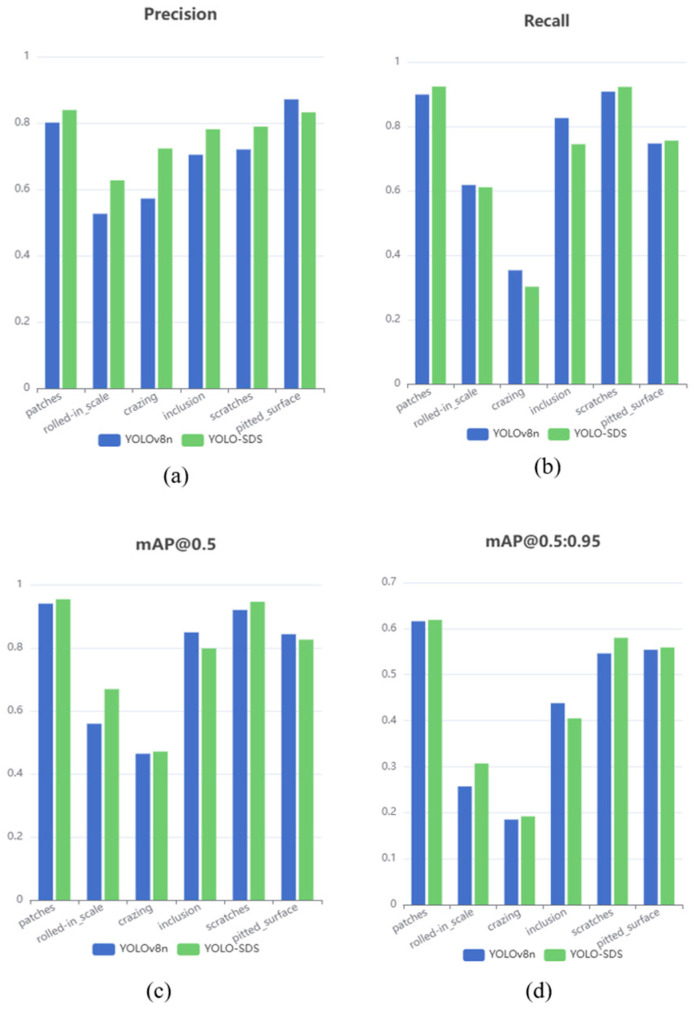
Detection outcomes for various types of defects. (**a**) Precision; (**b**) Recall; (**c**) mAP@0.5; and (**d**) mAP@0.5:0.95.

**Figure 9:**
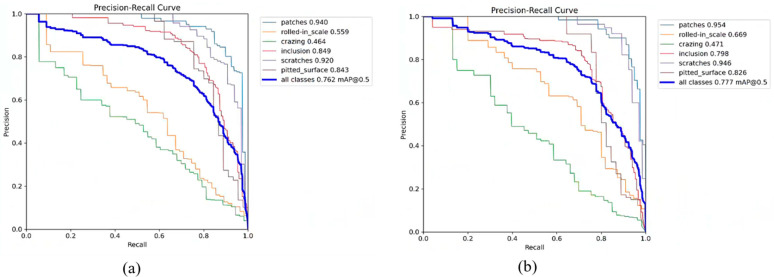
P–R curve of different kinds of defects. (**a**) YOLOv8n and (**b**) YOLO–SDS.

We also calculated the precision, recall, and mAP for the six types of strip steel surface defects in the detection results of YOLOv8n and YOLO-SDS and compared them. Subsequently, we plotted the rR curves for each type of defect, as shown in Figure 8 and Figure 9. In Figure 8 and Figure 9, it can be seen that our network shows improvements in precision, recall, and mAP for most categories of strip steel surface defects, and the overall mAP also increases. This demonstrates the superiority of our network in detecting strip steel surface defects.

**Figure 10:**
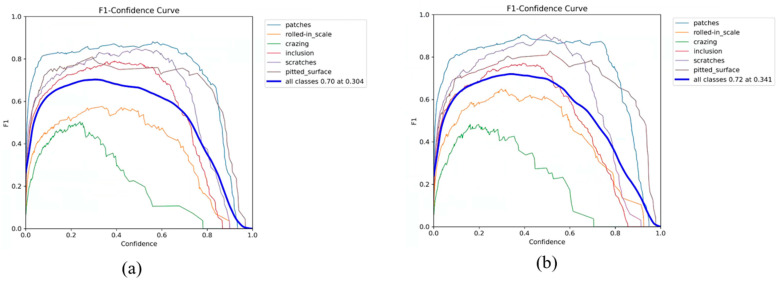
F1 score curve for different kinds of defects. (**a**) YOLOv8n and (**b**) YOLO-SDS.

The F1 score is a crucial performance metric for assessing the stability of a model; the higher the F1 score, the stronger the model’s performance. We calculated the F1 scores for YOLOv8n and YOLO-SDS and graphed the associated curves, as shown in Figure 10. In Figure 10, it is evident that our model achieves a higher F1 score, indicating that the YOLO-SDS model performs better.

**Figure 11:**
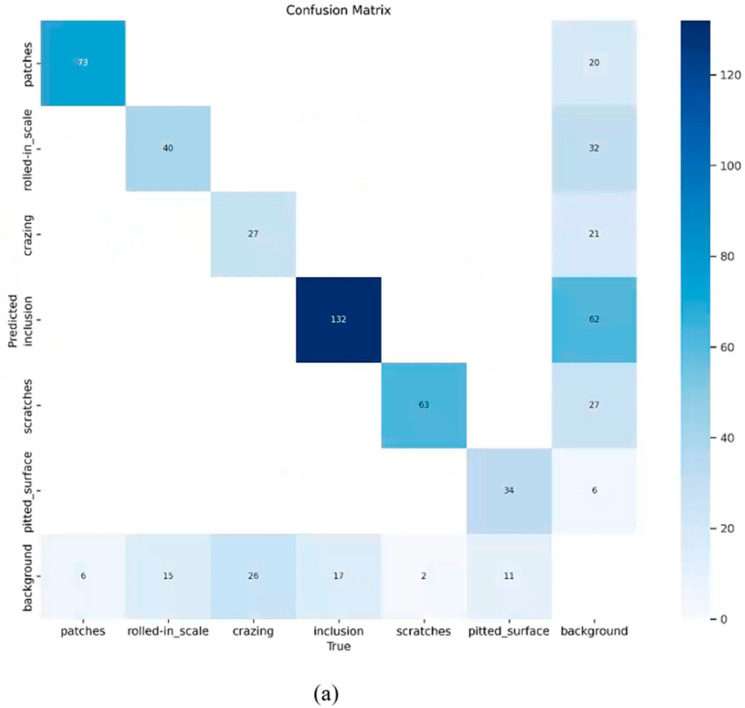

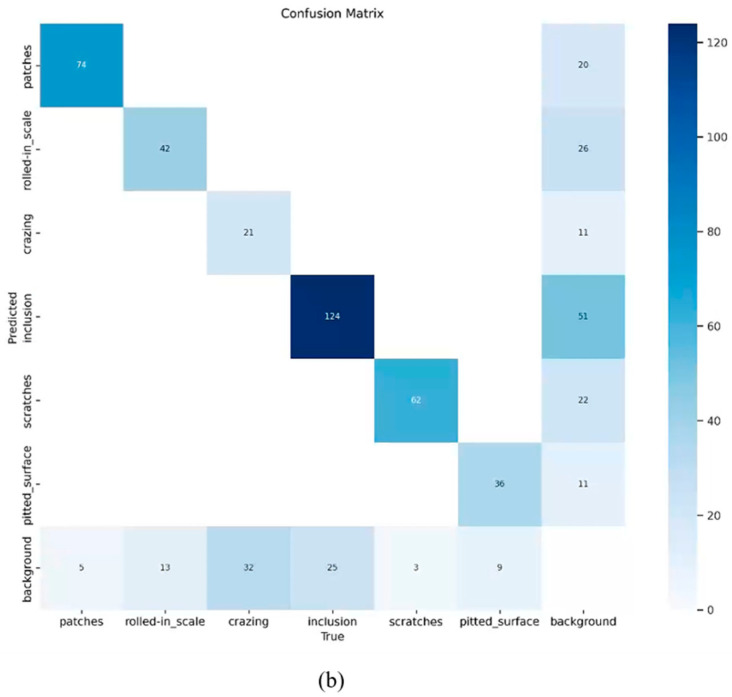
Confusion matrix for the validation set with defects of different classes. (**a**) Original and (**b**) YOLO-SDS.

Additionally, we used a confusion matrix to further assess the detection capabilities of the trained model. Figure 11 presents the experimental results. As seen in Figure 11, the false positives (FPs) and false negatives (FNs) for most defect categories are comparable to or possibly surpass the original data, indicating that the enhanced model achieves greater accuracy of classification.

### 4.5. Comparative Analysis with Other Approaches on NEU-DET

**Figure 12:**
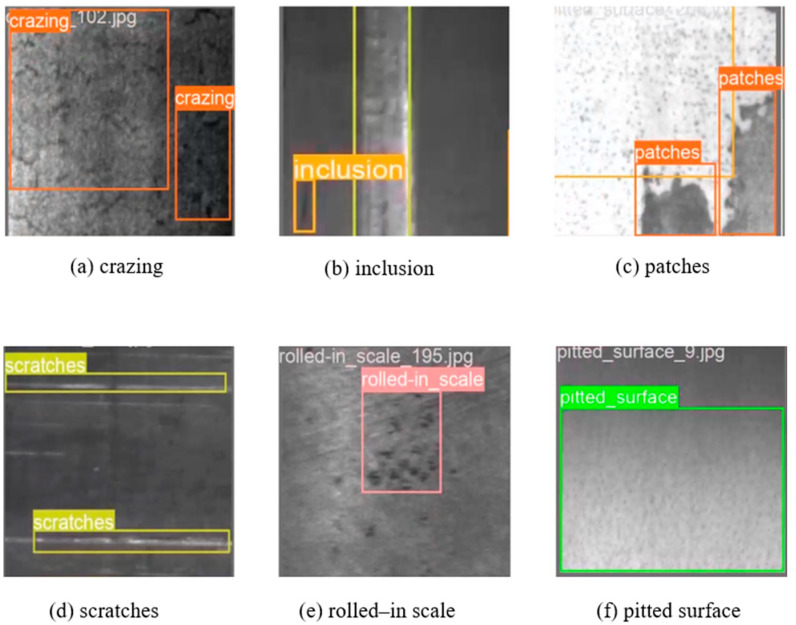
Detection outcomes on NEU-DET.

Figure 12 illustrates the detection outcomes of YOLO-SDS on the NEU-DET dataset. The results include various types of information such as prediction boxes and defect categories. As illustrated, both unclear defects and minor defects can be precisely detected.

We compared the proposed model with several widely used techniques to evaluate its effectiveness. The YOLO series includes YOLOv3-tiny, YOLOv5n, YOLOv6n, and YOLOv8n. Popular lightweight backbone networks used to replace the YOLOv8n backbone include MobileNetv4 [36], Fasternet, and GhostHGNetv2 [37], as well as other recent YOLO models. To ensure data reliability, the quantity of epochs was uniformly set to 200, and all models were experimented on the NEU-DET dataset. The comparison results with the YOLO-SDS network are shown in Table 1, Table 2 and Table 3. As indicated in Table 1, our proposed model demonstrated the best performance among the YOLO series, with significantly fewer parameters and the lowest computational cost. Table 2 demonstrates that our model surpasses other lightweight networks in both precision and mAP. Although the recall of YOLO-SDS is inferior to that of alternative models, our model has the fewest parameters. The inference efficiency of our model in Table 1 and Table 2 is average, but it still meets the requirements for real-time processing. In Table 3, the detection precision of Yolo-sd and MSC-Dnet is slightly higher than that of our proposed model, but their floating-point operations are far higher than our model. The other models are less than ours with respect to average accuracy and parameter count. Our proposed YOLO-SDS effectively balances detection accuracy and model complexity.

**Figure 13:**
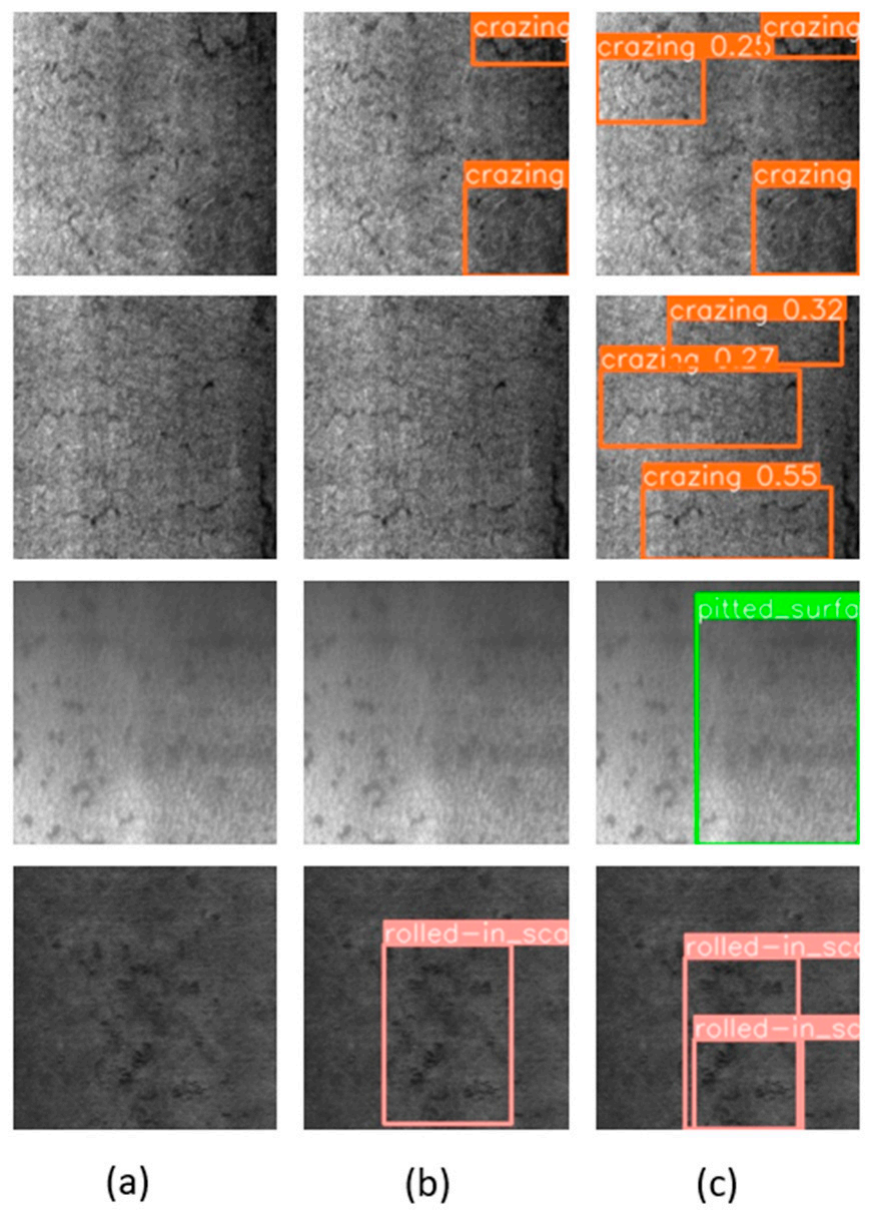
Visual comparison between YOLO-SDS and the original YOLOv8n. (**a**) Original; (**b**) YOLOv8n; and (**c**) YOLO-SDS.

To observe the performance improvement after model enhancement more clearly and intuitively, we provided visual images of defect detection by YOLO-SDS and YOLOv8n, as illustrated in Figure 13. The data in Figure 13 show that the original YOLOv8n model missed detection issues in the target detection task. For instance, the defect types in the second row and second column, as well as the third row and second column, were not detected. Additionally, the model exhibited missed detections in the first row and second column and the fourth row and second column. By comparing the detection results in the third column of Figure 13, it is clear that the enhanced algorithm effectively addresses the missed detection problem. Overall, the improved algorithm demonstrates its superiority in multiple aspects. It successfully addresses the issue of missed detections, significantly reducing the occurrence of defects being overlooked and ensuring the comprehensiveness of the detection process. The algorithm excels in enhancing detection accuracy, enabling more precise identification and classification of different types of defects, which further reduces the rate of false detections. These improvements validate the algorithm’s effectiveness, making it more reliable and practical for real-world applications.

### 4.6. Ablation Study

In order to assess the performance and efficiency of the proposed network, we conducted various experiments on the components of the network architecture, as detailed in Table 4. As shown in Table 4, when only StarNet is added, the model’s parameter quantity along with its complexity are significantly reduced; however, the mAP@0.5:0.95 also decreases. When both StarNet and SEAM are added, the mAP@0.5 decreases, and the parameter count and computational load further reduce, but the detection accuracy is insufficient. Adding both StarNet and C2f_DWR improves the mAP to a higher level while keeping the total parameters and the computational workload low. When both C2f_DWR and SEAM are added, the mAP improves, and the parameter count and computational load also decrease. Clearly, our model surpasses YOLOv8n in terms of the mAP while keeping a lower parameter count and reduced computational burden, achieving better lightweight performance. This suggests that YOLO-SDS strikes a more favorable harmony between detection performance and the model’s complexity.

### 4.7. Analysis of Failure Cases

**Figure 14:**
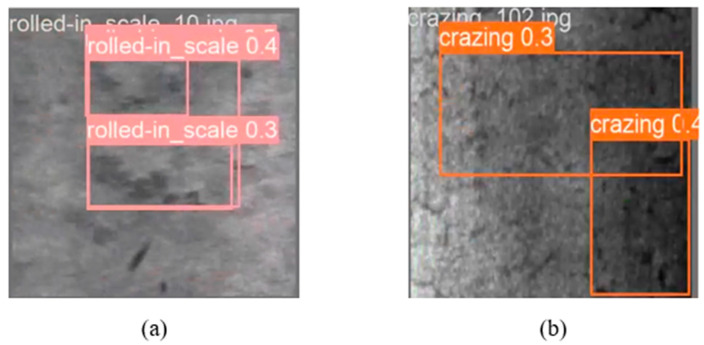
Some cases of detection failures. The defects are very blurry, making it difficult for the detector to detect them accurately. (**a**) rs and (**b**) cr.

In comparison with cutting-edge methods, YOLO-SDS is a highly competitive detector that delivers outstanding performance on the NEU-DET dataset. However, it is noteworthy that the mAP values for cracks and rolled-in scale are the lowest among all categories, indicating a relatively higher number of detection failures for these two defects. As shown in Figure 14, our model faces challenges in accurately identifying the defect categories and their locations. We infer that the visual characteristics of cracks and rolled-in scale may be more complex or less apparent, complicating the model’s ability to tell them apart from the background or other categories. These defects may exhibit a wide range of morphological variations or intricate texture details, increasing the detection difficulty. Cracks and rolled-in scale often present smaller or less conspicuous features in images, resulting in poor model performance when dealing with these small-scale features. If the image resolution is low or cracks and rolled-in scale take up a small portion of the image, the model may not effectively detect them. To address these issues, data augmentation can be applied to enhance the diversity of these two defect types. Alternatively, improving the model architecture or tuning the hyperparameters specifically for these two defect types can help enhance the model’s detection capability for these defects.

### 4.8. Generalization Experiments

**Figure 15:**
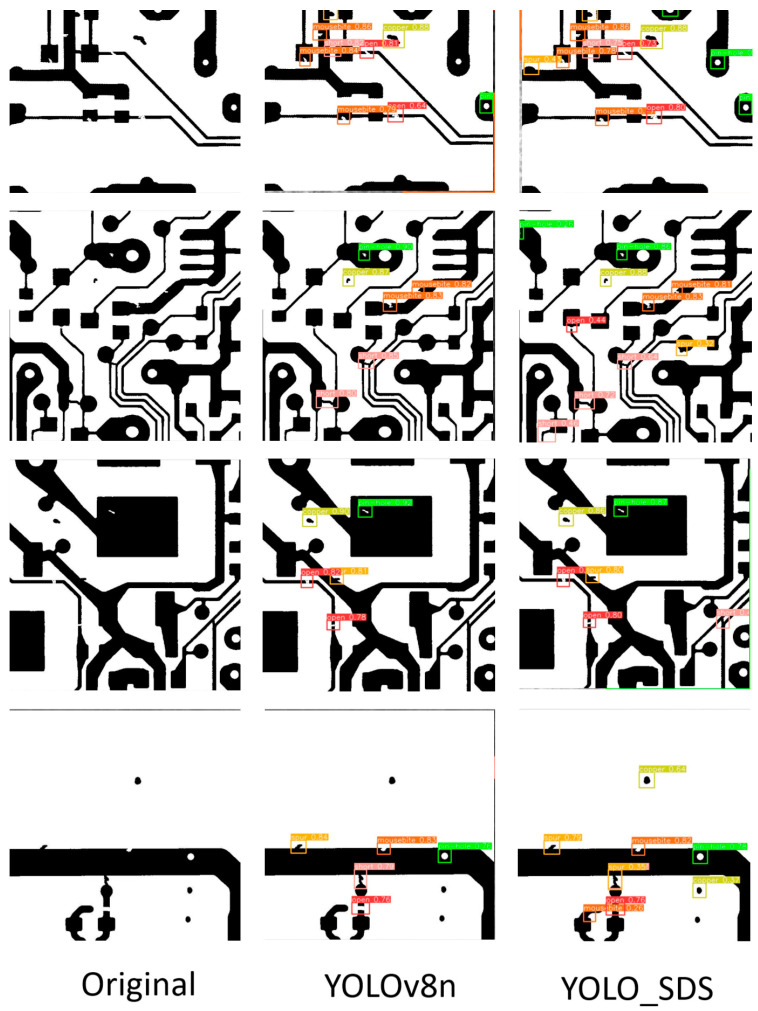
Comparative experimental results on the deepPCB dataset.

To further verify the stability and generalization of the improved algorithm presented in this paper, we compared the algorithm we proposed with several other algorithms. The deepPCB dataset contains six types of defects on the surface of PCB boards, including open circuit (open), short circuit (short), mouse bite (mousebite), missing hole (pin-hole), spur (spur), and spurious copper (copper), with a total of 1500 images. The deepPCB dataset is allocated between training and testing sets with a 9:1 distribution, resulting in 1350 images for training and 150 images for testing. The experimental results are shown in Table 5, and it can be observed that our proposed model performs excellently on this dataset, with an mAP value of 0.981. The number of parameters and FLOPs are the lowest among these methods. The modified model’s parameter count is 66% of YOLOv8n, while its mAP is only 0.6% lower than that of YOLOv8n. This indicates that our model is more lightweight while maintaining high detection accuracy. Although the inference speed of the model is reduced, it still meets the requirements of industrial defect detection. Compared with other defect detection methods in recent years, the model we proposed balances both detection accuracy and inference speed. Figure 15 shows the detection results of the YOLOv8n model and our proposed model on the DeepPCB dataset. It can be seen that many defects that were not detected by the YOLOv8n model were detected by our proposed model, which proves the superiority of our proposed model.

## 5. Conclusions

Drawing from YOLOv8, this paper presents a novel detector for steel surface defect identification, YOLO-SDS, by refining the backbone, neck, and head architecture. YOLO-SDS exhibits outstanding performance in terms of both accuracy and speed of detection. To achieve lightweight networking while maintaining accuracy, the StarNet module was integrated as a key element of the YOLO-SDS backbone. StarNet maps inputs to a high-dimensional nonlinear feature space through efficient Star Operations, similar to kernel methods, but without increasing network complexity, thus achieving efficient data processing and feature extraction. This significantly decreases the total parameter quantity in the network and computational burden. In the neck, a lightweight module, DWR, is introduced and combined with YOLOv8’s feature extraction module, C2f. By introducing gaps between convolutional kernels, the receptive field is expanded, allowing the acquisition of a wider range of contextual information. In the head, we incorporate the occlusion-aware attention mechanism, SEAM, which enhances the model’s accuracy and generalization ability, significantly improving its target detection performance in complex environments. Experiments on the NEU-DET and DeepPCB datasets were carried out to assess its robustness and generalization capabilities. In comparison with cutting-edge techniques, YOLO-SDS achieved a 77.7% mAP and 1.79 M Params on NEU-DET, demonstrating that our proposed model is highly competitive within steel surface defect detection networks. These experimental results confirm that YOLO-SDS meets the requirements for both accuracy and speed of detection. However, the performance of YOLO-SDS needs improvement in some blurry and minor defects, such as cracks and rolled-in scale. In future research, we plan to improve our network by incorporating image preprocessing techniques or adopting more robust backbones. Additionally, we will optimize the structure to further improve detection accuracy while maintaining a lightweight model.

## Figures and Tables

**Table 1:** Performance comparison on the NEU-DET dataset (mainly in the YOLO series).

Method	Precision	Recall	F1	mAP@0.5	Params (M)	FLOPs (G)	FPS
YOLOv3-tiny	0.658	0.676	0.667	0.704	12.13	18.9	244
YOLOv5n	0.676	0.724	0.699	0.755	2.5	7.1	195
YOLOv6n	0.721	0.729	0.725	0.769	4.23	11.8	174
YOLOv8n	0.699	0.725	0.712	0.762	3	8.1	190
Ours	0.765	0.71	0.736	0.777	1.97	5.3	168

**Table 2:** Performance comparison on the NEU-DET dataset (mainly on the lightweight backbone series).

Method	Precision	Recall	F1	mAP@0.5	Params (M)	FLOPs (G)	FPS
MobileNetv4	0.657	0.754	0.702	0.775	5.7	22.5	188
Fasternet	0.732	0.736	0.734	0.776	4.17	10.7	153
GhostHGNetv2	0.73	0.714	0.722	0.767	2.31	6.8	201
Ours	0.765	0.71	0.736	0.777	1.97	**5.3**	168

**Table 3:** Comparison of the performance of other models on NEU-DET in the past two years.

Method	Params (M)	FLOPs (G)	mAP	cr	in	pa	ps	rs	sc
Yolo-sd [38]	—	84.4	0.823	0.544	0.846	0.936	0.86	0.775	0.956
GDCP-YOLO [39]	2.8	—	0.758	—	—	—	—	—	—
Trident-LK [40]	9.21	2.22	0.769	0.388	0.812	0.903	0.863	0.688	0.959
SDD-YOLO [41]	3.4	6.4	0.761	0.581	0.881	0.949	0.934	0.617	0.913
MSC-Dnet [42]	34.1	78	0.794	0.424	0.845	0.943	0.915	0.716	0.920
TD-Net [43]	7.06	—	0.768	—	—	—	—	—	—
Fast-RCNN [44]	137.09	370.2	0.759	—	—	—	—	—	—
Ours	1.97	5.3	0.777	0.471	0.798	0.954	0.826	0.669	0.946

**Table 4:** Comparison of defects detection performance of different modules.

Number	StarNet	SEAM	C2f_DWR	Params (M)	FLOPs (G)	mAP@0.5	mAP@0.5:0.95
N1				3	8.1	0.762	0.433
N2	√			2.21	6.5	0.764	0.421
N3	√	√		2.02	5.4	0.756	0.434
N4	√		√	2.16	6.4	0.774	0.428
N5		√	√	2.76	6.9	0.766	0.438
N6	√	√	√	1.97	5.3	0.777	0.444

**Table 5:** Performance comparison on the deepPCB dataset.

Method	Precision	Recall	mAP@0.5	Params (M)	FLOPs (G)	FPS
YOLOv3-tiny	0.864	0.833	0.906	12.1	18.9	246
Faster-RCNN [44]	—	—	0.942	4.17	33.6	21.5
YOLOv5 [45]	0.970	0.956	0.971	46.1	108.5	—
YOLOv8n	0.975	0.948	0.987	3	8.1	193
SSD [45]	—	—	0.959	24.3	116.4	64
FCOS [45]	0.939	0.963	0.971	32.1	68.3	—
GPP-AP [46]	—	—	0.971	—	—	62
Ours	0.943	0.949	0.981	1.97	5.3	168

## Data Availability

The data presented in this study are available on request from the corresponding author.
